# Influence of Different Cationic Polymer-Based Micelles on the Corneal Behavior and Anti-Cataract Effect of Diosmetin

**DOI:** 10.3390/pharmaceutics17030302

**Published:** 2025-02-25

**Authors:** Jing Zhang, Min Zha, Anping Wan, Satya Siva Kishan Yalamarty, Nina Filipczak, Xiang Li

**Affiliations:** 1 National Pharmaceutical Engineering Center for Solid Preparation of Chinese Herbal Medicine, State Key Laboratory for the Modernization of Classical and Famous Prescriptions of Chinese Medicine, Key Laboratory of Modern Preparation of TCM, Ministry of Education, Jiangxi University of Chinese Medicine, Nanchang 330006, China; jing.zhang@jxutcm.edu.cn (J.Z.); zhamin@jxutcm.edu.cn (M.Z.); wananping@jxutcm.edu.cn (A.W.); 2 China Resources Jiangzhong Pharmaceutical Group Co., Ltd., Nanchang 330004, China; 3 Center for Pharmaceutical Biotechnology and Nanomedicine, Northeastern University, Boston, MA 02115, USA; yalamarty.s@northeastern.edu (S.S.K.Y.); nin.filipczak@northeastern.edu (N.F.)

**Keywords:** diosmetin, cationic polymer, micelles, cataract, PAMAM, trimethyl chitosan

## Abstract

**Background** Despite many studies on polymer-incorporated nanocarriers for ophthalmic drug delivery, few have thoroughly explored the relationship between coating composition and performance. This study aimed to evaluate the effects of three commonly used cationic polymers—distearoyl phosphatidylethanolamine-polyethylene glycol 1000-poly(amidoamine) (DSPE-PEG1000-PAMAM), trimethyl chitosan (TMC), and (2,3-dioleoyloxypropyl) trimethylammonium chloride (DOTAP)—on the corneal behaviors and anti-cataract efficacy of diosmetin (DIO)-loaded micelles (D-M-P, D-M-T, and D-M-D, respectively). **Methods** The DIO-loaded micelles were prepared using the thin-film dispersion method and incorporated with the three polymers through hydrophobic interactions and electrostatic adsorption. Structural characterization was demonstrated by TEM imaging and particle size analyzer. In vitro release behavior was detected by the dialysis method. Cell viability of D-M-P, D-M-T, and D-M-D on L929 cells was detected by CCK-8 assays, with cellular uptake performed using coumarin 6 as the fluorescence indicator. Precorneal retention behaviors of these three vesicles were observed by In Vivo Imaging System. Transcorneal permeability was determined by modified Franz diffusion method and the permeation routes of the vesicles are investigated. Selenite-induced cataract model was established. The anti-cataract effects of three different DIO-loaded micelles were evaluated by the observation of lens opacity and antioxidant enzyme activities. Eye Irritation of the DIO in different preparations was estimated using the Draize test, along with H&E staining of the corneas. **Results** Structural characterization of DIO-loaded micelles revealed that the vesicles were spherical, with a uniform size distribution of around 28 nm, a similar surface potential of approximately 6.0 mV, and a high DIO entrapment efficiency of about 95%. Compared to the DIO suspension, all three formulations exhibited a significant sustained-release effect. They showed no signs of irritation and demonstrated increased IC50 values in L929 cells, indicating improved biocompatibility. Cellular uptake in human lens epithelial cells (HLECs) was assessed using confocal laser scanning microscopy. C-M-T displayed the highest fluorescence signals, with a cellular internalization 3.2 times greater than that of the solution group. Both C-M-T and C-M-P enhanced vesicle retention on the corneal surface by at least 47.8% compared to the Cou-6 solution. Furthermore, TMC facilitated the paracellular transport of vesicles into the deepest layers of the cornea and delivered DIO across the cornea, with a *P*_app_ value 3.11 times and 1.49 times those of D-M-D and D-M-P, respectively. In terms of therapeutic efficacy, D-M-T demonstrated the most significant attenuation of lens opacity, along with enhanced antioxidant enzyme activities and inhibition of lipid peroxidation. **Conclusion** The modification of micelle vesicles with different cationic polymers significantly influences their performance in ocular drug delivery. Among the tested formulations, D-M-T stands out due to its multiple advantages, including enhanced transcorneal drug delivery, therapeutic efficacy for DIO, and safety, making it the most promising candidate for ophthalmic applications.

## 1. Introduction

Cataracts, the leading cause of global blindness, impose a heavy socioeconomic burden, with surgical treatment remaining the primary but imperfect solution due to the risks of complications and high costs [1,2,3,4,5]. Topical ophthalmic formulations are preferred and account for 90% of the marketed ocular formulations owing to their non-invasiveness, convenience, and patient compliance [6]. However, they face challenges, including poor bioavailability and efficacy, which are attributed to distinct physiological barriers and the anatomical structure of the eye [7]. To address these limitations, cationic polymers have emerged as promising candidates for enhancing ocular drug delivery through mucoadhesion and transcorneal transport [8,9]. Among them, trimethyl chitosan (TMC), poly(amidoamine) dendrimers (PAMAM), and 1,2-dioleoyl-3-trimethylammonium-propane chloride (DOTAP) salt are widely studied but rarely systematically compared for their distinct mechanisms and performance.

TMC, a positively charged derivative of the quaternization of chitosan, binds to the polyanionic surface of the ocular mucosa, known as the mucin layer, via hydrogen bonding and ionic interactions. In the end, it increases the mucoadhesive ability of the formulation [10]. Moreover, TMC transiently opens tight junctions, by triggering a rearrangement of cytoskeletal F-actin and the tight junction protein ZO-1, to facilitate paracellular pathways [8,11]. PAMAM is a positively charged dendrimer with a highly symmetrical structure and numerous surface amino groups. It is water-soluble and non-immunogenic, and its biocompatibility can be improved by polyethylene glycol modification [12]. Studies have shown that the positive charges on PAMAM enhance drug corneal permeability and prolong drug retention in the cornea, related to the generation of PAMAM dendrimers [13]. Higher-generation PAMAM dendrimers can incorporate anionic unilamellar vesicles and more strongly affect cell and lipid membranes, altering cell permeability [14,15]. DOTAP works as a cationic surfactant to increase the stability of the genetic drug under infinite dilution mimicking ocular administration [16,17], and it has also been reported to be incorporated into bilayers of lipids in liposomal formation to engage with the mucous layer that coats the cornea [18].

These three polymers were explored for the link between lipid composition and its performance. A better understanding of this relationship is crucial for optimizing the design and formulation of ocular drug delivery systems. To achieve this goal, this study chose diosmetin (DIO), an essential active component of chrysanthemum, to comprehensively evaluate their performance both in vitro and in vivo.

DIO was reported to mitigate the damage inflicted on lens epithelial cells (LECs) by decreasing the activation of the MAPK pathway triggered by oxidative stress [19,20]. Due to its antioxidant and anti-apoptotic characteristics, it can protect tissues against damage under various pathological circumstances. Since oxidative stress is a key factor in cataract formation in relation to the maintenance of the redox balance [21], DIO has the potential for anti-cataract efficacy. However, due to its chemical structure, the inevitable shortcomings of DIO involve low solubility and poor membrane permeability; therefore, its application in eye diseases still requires additional exploration.

In this study, we conducted a comprehensive investigation with two main focuses. First, by comparing the performance of cationic polymer-based micelles formed with DSPE-PEG-PAMAM, DOTAP, and TMC, we aim to gain insights into the influence of each polymer’s inherent characteristics on micelle performance. This study also pioneers the in-depth exploration of the anti-cataract effect of different DIO-loaded polymer-based micelles. These formulations were validated through in vitro characterization (size, zeta potential, drug release), cellular uptake assays, transcorneal permeability studies, and in vivo efficacy tests in selenite-induced cataract models. This work prioritizes mechanistic insights into polymer-driven delivery, ultimately contributing to the development of more effective ophthalmic formulations for cataract treatment.

## 2. Materials and Methods

### 2.1. Materials

DIO (purity > 98.0%) and coumarin 6 (Cou-6, purity > 98.0%) were purchased from Shanghai Aladdin Bio-Chem Technology Co., Ltd. (Shanghai, China). The 1,2-dioleoyl-sn-glycero-3-phosphoethanolamine-conjugated poly(ethylene glycol) 2000 (DSPE-PEG2000), 1,2-dioleoyl-3-trimethylammoniumpropane (DOTAP) and 1,2-distearoyl-sn-glycero-3-phosphoethanolamine-N-(carboxy(polyethylene glycol)-1000, NHS ester (DSPE-PEG1000-NHS) were purchased from Xi’an Ruixi Biological Technology Co., Ltd. (Xi’an, China). PAMAM (G4.0) was purchased from Sigma-Aldrich, Co. (St. Louis, MO, USA). DSPE-PEG1000-PAMAM (G4.0) was self-synthesized (see [22]). D-α-tocopherol polyethylene glycol 1000 succinate (TPGS) was purchased from Dalian Meilun Biotechnology Co., Ltd. (Dalian, China). TMC (quaternization = 33%) was purchased from Changzhou Shijia Biomedicine Technology Co., Ltd. (Changzhou, China).

Human lens epithelial cells (HLEC) and L929 cells were obtained from Beijing Dingguo Changsheng Biotechnology Co., Ltd. (Beijing, China). Cells were grown in Dulbecco’s modified Eagle medium/Roswell Park Memorial Institute 1640, respectively, with 10% fetal bovine serum (FBS) (Yeasen Biotechnology (Shanghai) Co., Ltd., Shanghai, China) and 1% penicillin/streptomycin (Beijing Solarbio Science & Technology Co., Ltd., Beijing, China) at 37 °C in a humidified atmosphere of 5% CO_2_.

Male albino New Zealand rabbits (weight: 2.0~2.5 kg; free of clinically observable abnormalities) were obtained from Henan SCBS Biotechnology Co., Ltd. (Anyang, China; SCXK (Yu) 2024-0001). Sprague–Dawley rat pups (age: 11 days; male and female; weight: 18–22 g) raised with their mothers were obtained from Hunan SJA Laboratory Animal Co., Ltd. (Changsha, China; SCXK (Xiang) 2021-0002). Before the experiment, they were housed under standard conditions (temperature: 22 ± 2 °C; humidity: 40–60% relative humidity; 12 h light/dark cycle) and fed with a standard pellet diet and water ad libitum. The protocols for the use and care of animals were approved by the Jiangxi University of Chinese Medicine (Approval No. JZLLSC20240586).

### 2.2. Preparation of Cationic Polymer Micelles

DIO-loaded micelles (D-M) were prepared by thin film dispersion. DIO, TPGS, and DSPE-PEG2000 were dissolved in ethanol to form a lipid solution in a mass ratio of 0.5:7:3 and transferred to a rotary steaming flask. The uniformly dispersed thin lipid film was formed by rotatory evaporation at 43 °C, 30 rpm, and 140 mbar for 1 h and then hydrated with a 5% glucose solution for 1 h to obtain D-M. Then, a 2% TMC solution was incubated with D-M in a volume ratio of 1:1 to obtain D-M coated with TMC (D-M-T).

The lipid film was obtained as described above, 0.140 mmol/L of DSPE-PEG1000-PAMAM aqueous solution was hydrated, and then polymer micelles were obtained with DSPE-PEG1000-PAMAM as cationic material (D-M-P).

DOTAP was added to the lipid solution of DIO, TPGS, DSPE-PEG2000 in a mass ratio of 0.5:7:3:5 for DIO:TPGS:DSPE-PEG2000:DOTAP, transferred to a rotary steaming flask. The uniformly dispersed thin lipid film was formed by rotatory evaporation at 43 °C, 30 rpm, and 140 mbar for 1 h and then hydrated with a 5% glucose solution for 1 h to polymer micelles with DOTAP as cationic materials (D-M-D).

For visualization of micellar vesicle penetration through the corneas, the fluorescence marker Cou-6 was used. The incorporation of Cou-6 into micelles was conducted as indicated above, except that the Dio was replaced by Cou-6 to obtain the lipid solution, to obtain Cou-6-loaded TMC-coated micelles (C-M-T), DSPE-PEG1000-PAMAM-modified micelles (C-M-P) and DOTAP-modified micelles (C-M-D), where the Cou-6 concentration was 10 μg/mL.

### 2.3. Characterization of Cationic Polymer Micelles

#### 2.3.1. Particle Size, Potential, and Morphology

The particle size and Zeta potential of D-M-T, D-M-D, and D-M-P were determined using a particle size analyzer (Nano-ZS ZEN3690, Malvern Instruments, Malvern, UK). The appropriate micelle suspension was dropped on the copper carbon film net, dried naturally, and stained with 2% phosphotungstic acid, and then the morphology was observed by transmission electron microscope (TEM) (JEM-2100, JEOL, Tokyo, Japan, the point resolution is 0.19 nm).

#### 2.3.2. Encapsulation Efficiency

The unencapsulated DIO in micelles or liposomes were separated from the encapsulated DIO by ultracentrifugation [23]. Briefly, 0.5 mL of suspensions of D-M-D, D-M-T, and D-M-P were placed in the ultrafiltration tube separately and centrifuged at 3000 revolutions per minute (rpm) for 30 min. As a result, the free drug in the supernatant was diluted and determined by HPLC as W1 in the Diamonsil C18 column (250 mm × 4.6 mm, 5 μm). The mobile phase was water-methanol (volume ratio 33:67, *v*/*v*), the column temperature was 30 °C, the detection wavelength was 346 nm, the flow rate was 1.0 mL/min, and the injection volume was 10 μL. Next, 0.5 mL of suspension was diluted with 5.0 mL of methanol to determine the total drug concentration (W2) by HPLC. The encapsulation efficiency calculation formula was as follows: EE (%) = W1/W2 × 100.

#### 2.3.3. In Vitro Release Experiment

The in vitro release behavior of DIO from different preparations was detected using the dialysis method [24]. Briefly, the appropriate volume of suspensions, D-M-D, D-M-T, and D-M-P, with the same concentration as DIO, was placed in a dialysis bag (MW cutoff: 20 kDa) and placed in 110 mL 0.3% Tween 80-Ringer aqueous solution. The experiment was conducted at 37 °C under stirring at 250 rpm. The free DIO suspension (free DIO) was prepared as a control and subjected to the same protocol. At decided time intervals of 0.5, 1, 2, 4, 8, 12, and 24 h, the sample was collected from the releasing medium, and the same amount of prewarmed blank releasing medium was added. The concentration of DIO in the samples was measured using the HPLC condition described above. The cumulative release rate of DIO from preparations was calculated as follows:

(1) $$\mathrm{Cumulative}\;\mathrm{release}\;\mathrm{rate}\;\mathrm{of}\;\mathrm{DIO}\;(\%)=\frac{{DIO}_{t}-{DIO}_{i}}{{DIO}_{t}}\times 100$$

where DIO_t_ was the total concentration of DIO at t = 0 and DIO_i_ was the concentration of DIO in dialysis bags at different time points.

### 2.4. Cell Viability Assay

Cell viability was measured by the CCK-8 assay (MA0218, Dalian Meilun Biotechnology Co., Ltd., Dalian, China) according to the manufacturer’s protocol. L929 cells were inoculated in a 96-well plate at a cell density of 2 × 10^4^/well and cultured to cell adhesion at 37 °C and 5% CO_2_. Subsequently, the culture medium was replaced with a serum-free medium for 12 h of starvation treatment. The cells were then exposed to varying concentrations of DIO dissolved in DMSO, D-M-D, D-M-T, and D-M-P, all within SFM and incubated for 24 h. The medium was discarded, with 100 μL 10% CCK-8 reagent in each well for 2 h. A microplate reader (SpectraMax ^®^i3, Molecular Devices, San Jose, CA, USA) was used to test the absorbance of each experimental well at 450 nm, and the IC50 was calculated using GraphPad Prism 8.0 software.

### 2.5. Cell Uptake Assay

Cellular uptake of the nanoparticles was measured by confocal laser scanning microscopy (CLSM). HLEC cells were inoculated on Lab-Tek^®^ chamber slides at 2 × 10^4^ cells/well and cultured at 37 °C and 5%CO_2_ until adherent. C-M-D, C-M-T, and C-M-P micelles (Cou-6 concentration = 10 μg/mL) were prepared by using fluorescent probe Cou-6 instead of DIO. Cells were incubated with C-M-D, C-M-T, and C-M-P for 2 h with Cou-6 solution as the control. After incubation, cells were washed with PBS three times and stained with DAPI for 10 min. After 3 washes with PBS, the slides were sealed and the fluorescence distribution in the cells was observed by CLSM analysis (TCS SP8, Leica, Wetzlar, Germany).

### 2.6. Precorneal Retention Ability

The rabbits were anesthetized by intraperitoneal injection of 1% pentobarbital at a dose of 40 mg/kg. The rabbits were randomly divided into four groups, and 10 μL Cou-6 solution, C-M-D, C-M-T, and C-M-P micelles were dropped into the rabbit conjunctiva sac. At 0.5, 2.5, 4.5, 6.5, 9.5, 11.5, 14.5, 16.5, and 19.5 min, drug retention was observed by In Vivo Imaging System (Lumina XRMS Series III, PerkinElmer, Springfield, IL, USA).

### 2.7. Transcorneal Permeability

#### 2.7.1. DIO Transcorneal Penetration

The modified Franz diffusion method was applied [25,26]. Rabbit cornea tissues were positioned between the two compartments of a Franz cell with a diffusional surface area of 0.5 cm^2^, with the corneal side facing upwards, toward the donor chamber, and with the other side in contact with the receptor medium. A total of 6.5 mL of preheated Ringer’s solution (GBR) mixed 1:1 was added to the receptor cell. DIO suspension and the micelles solution of D-M-D, D-M-T, and D-M-P of 0.5 mL were added to the donor cell. The permeation study was carried out for 4 h at 34 ± 0.5 °C under continuous stirring (200 rpm) to maintain the sink condition. One milliliter of the sample was removed from the receptor cell for determination at 10, 20, 30, 60, 60, 90, 120, 150, 180, and 240 min, respectively, and an equal volume of fresh medium was added. The DIO in the samples was analyzed by HPLC as described above, and the cumulative permeability (*Q_n_*), apparent permeability coefficient (*P_app_*), and steady-state flow rate (*J_ss_*) were calculated as follows:

(2) $$Q_{n}=V_{0}\times C_{n}+V\cdot \sum_{i=1}^{n-1}C_{i}$$

where *V*_0_ is the volume of GBR solution in the receiving cell, *C_n_* is the DIO concentration measured at the time point t, *V* is the sampling volume and *C_i_* is the DIO concentration at the previous time point.

(3) $$P_{app}=\frac{∆Q}{60\cdot A\cdot ∆t\cdot C_{0}}$$

*Q*/*t* is the slope of the cumulative transmittance curve *Q_n_* and time, *A* is the effective diffusion area, and *C*_0_ is the initial administration concentration.

(4) $$J_{ss}=C_{0}\cdot P_{app}$$

#### 2.7.2. Micellar Vesicle Penetration

The experimental protocols were like those described above, except that the 0.5 mL C-M-D, C-M-T, and C-M-P micelles were placed in the receiving pool and the Cou-6 solution was used as a control. The cornea was removed at 2 and 4 h, respectively, washed, and laid on a confocal plate. The objective magnification of the confocal laser scanning microscope was 40×, the excitation wavelength of Cou-6 was 467 nm, the emission wavelength was 502 nm, and each frame was 5 μm for 3D scanning. The corneas incubated with C-M-T for 2 h were scanned every 5 μm depth from the cornea’s surface, and the XY plane scan was performed to observe the transcorneal pathway.

### 2.8. Evaluation of Anticataract Efficacy

Eleven-day-old suckling SD rats were randomly divided into the model group, blank control group, D-M-D group, D-M-T group, and D-M-P group. Except for the blank control group, all rats were subcutaneously injected with a 19 μmol/kg sodium selenite saline solution to induce cataracts [26]. Micellar suspensions were administered topically on the ocular surface with 15 μL administered per eye, 3 times a day for 7 days, whereas the rats in the model group and the blank control group were treated with 15 μL saline. Lens changes in the rat were recorded using a KJ5S3 handheld slit lamp microscope (Suzhou Kangjie Medical Inc., Suzhou, China) every day. After the last day of medication, the lens of each group was removed and milled with normal saline to obtain 10% weight lens homogenates. The supernatant was obtained by centrifugation lasting 15 min at 4 °C and 2500 rpm. The activity of catalase (CAT), the content of superoxide dismutase (SOD), and malondialdehyde (MDA) were detected according to the manufacturer’s instructions for the assay kits (Beyotime Biotechnology (Shanghai) Co., Ltd., Shanghai, China).

### 2.9. Eye Irritation

In vivo evaluation of the formulations tested for possible ocular irritation potential was conducted following the low-volume eye test procedure, which is a modification of the Draize test [27]. Rabbits with healthy eyes were divided into three groups. By self-control method, 40 μL D-M-D, D-M-T, D-M-P micelles were added to the corresponding right eye, and the same amount of normal saline was added to the left eye (3 times/day, for 7 days). Each time before and after administration, the conjunctiva, cornea, and iris of rabbits were observed for hyperemia, edema, and secretion. The ocular irritation index was calculated to indicate the irritant degree (See Table S1 in Supplementary Materials) [27,28,29].

After observation, rabbit eyeballs were removed, cornea, conjunctiva, and iris tissues were isolated, fixed with 4% PFA, dehydrated, embedded, sliced, stained with hematoxylin and eosin, and observed under a microscope.

### 2.10. Statistical Analysis

ImageJ 1.54 and GraphPad Prism 9.0 software were employed for statistical analysis and graph generation. All results are presented in the form of the mean ± standard deviation (SD). To assess significance, the t-test and the one-way analysis of variance were used, and statistically significant differences are indicated by *p*-values < 0.05.

## 3. Results and Discussion

### 3.1. Physicochemical Characterization of DIO-Loaded Preparations

**Figure 1:**
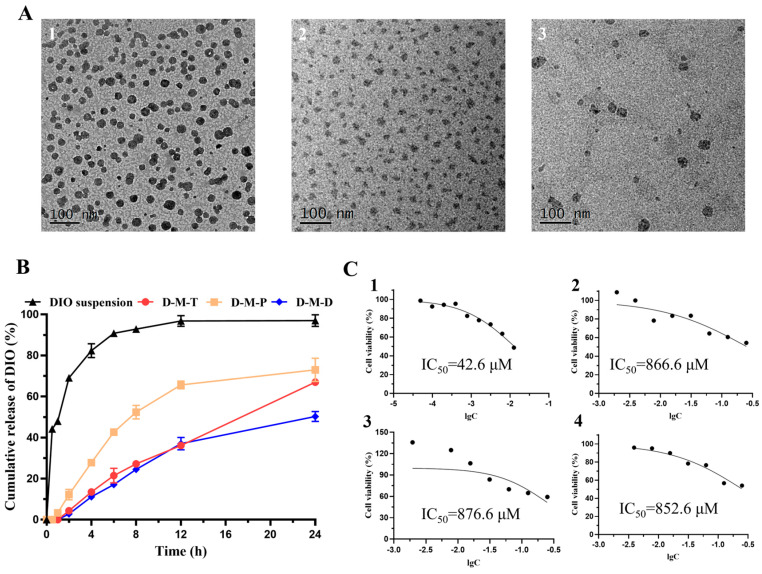
(**A**) TEM images of D-M-T (1), D-M-P (2), and D-M-D (3) (Bar = 100 nm); (**B**) in vitro release of DIO from different preparations at scheduled time points (n = 3); (**C**) cell viability of different concentrations of DIO (1), D-M-T (2), D-M-P (3), D-M-D (4) on L929 cells.

The particle sizes of D-M-D, D-M-T, and D-M-P were 28.42 ± 0.11 nm, 29.28 ± 0.48 nm, and 24.95 ± 0.15 nm, respectively. The potentials were 6.01 ± 1.14 mV, 6.46 ± 0.06 mV, and 6.19 ± 0.12 mV, respectively. The EE% encapsulation rates of DIO in D-M-D, D-M-T, and D-M-P were 97.46 ± 0.35%, 94.90 ± 0.41% and 94.78 ± 0.43%, respectively. According to the TEM scan findings, the morphology of the three micelles was regular and the size distribution was uniform (Figure 1A).

PAMAM is a cationic dendrimer with ammonia at its core [30]. When DSPE-PEG1000-PAMAM and phospholipids jointly form the structure of the micelles, positive charges are introduced to the surface of the electrically neutral micelles, causing the micelles to be overall positively charged. DOTAP is the most frequently used cationic lipid. The positively charged DOTAP can uniformly blend with the lipids and boosts the fluidity of the lipid bilayer to effectively hold large hydrophobic drugs within the lipid regions [31]. When it inserts into the micelle membrane, it can endow the micelles with positive charges [32]. Its positive charge imparts better solubility to the entire system and enables it to bind to negatively charged agents. The introduction of methyl groups onto the nitrogen atoms in TMC chains leads to the TMC chains displaying positive zeta potential. The nitrogen atoms attached to the TMC backbone can be protonated, thereby strengthening the net positive charge. Compared with chitosan, the permanently positive charge carried by the protonated amino groups of TMC serves as the driving force for its high solubility in water and other physiological fluids [33,34]. Therefore, with the incorporation of the three cationic polymers, D-M-D, D-M-T, and D-M-P displayed positive zeta potential.

From the physicochemical properties of D-M-D, D-M-T, and D-M-P, this characteristic equips them with similar physicochemical properties, a uniform particle size, positive zeta potential, and high EE%, ensuring a more valid assessment of the impact of the composition of different polymers on the in vivo processes and DIO effectiveness.

### 3.2. In Vitro Release

As shown in Figure 1B, compared to the DIO suspension, DIO in D-M-D, D-M-T, and D-M-P micelles had a slower rate of drug release, and there was no sudden release of the drug. The cumulative release rate of DIO in D-M-D, D-M-T, and D-M-P micelles at 12 h was 34.9 ± 3.0%, 35.3 ± 1.2%, and 64.4 ± 1.9%, respectively. The cumulative release of DIO suspension was almost 85% at 4 h and was completely released at 12 h, which indicated that the morphology of the three micelles was maintained within 4 h (drug release rate < 30% of total DIO), with a slow-release effect, which was beneficial to eye drug delivery.

### 3.3. Cytotoxicity

The CCK-8 assay was conducted to determine the cytotoxic capacity of the D-M-D, D-M-T, and D-M-P micelles in L929 cell lines with different concentrations. The toxicity of DIO was significantly reduced by the preparation of the above three types of micelles, and there was no obvious toxicity at different drug concentrations (Figure 1C). This may derive from the entrapment of DIO in the micellar vesicles and indicates the safe application of these polymers at the concentration used for eye drops.

### 3.4. Cellular Uptake

**Figure 2:**
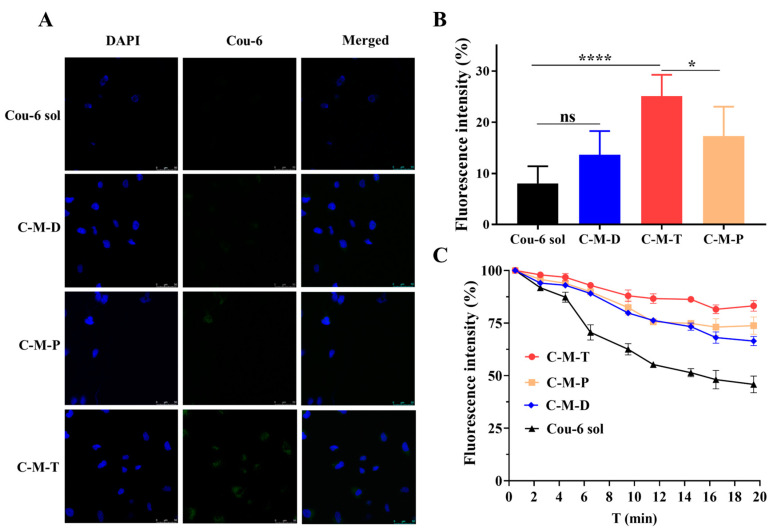
(**A**) Cellular uptake of vehicles in HLECs after incubation with different preparations (Bar = 50 μm); (**B**) intracellular fluorescence intensity of HLECs after incubation with different preparations (*, *p* < 0.05; ****, *p* < 0.0001; ns, not significantly); (**C**) precorneal fluorescence signals of each group in rabbit corneas under IVIS imaging at different time points within 20 min.

The fluorescent dye Cou-6 was used as a probe to track the nanoparticles. Thus, C-M-D, C-M-T, and C-M-P micelles were prepared using the fluorescent probe Cou-6 instead of DIO. After 4 h, the four groups of cells showed a certain capacity to absorb drugs, where DAPI stained the nucleus blue and fluorescence green, indicating the distribution of vesicles in the cells. CLSM analysis confirmed that the fluorescence intensity of the C-M-D, C-M-T, and C-M-P groups was 1.6, 3.2, and 2.0 times higher, respectively, than the Cou-6 solution group (Figure 2A,B). Compared to the Cou-6 solution group, intracellular uptake of C-M-P and C-M-T was significantly enhanced. The results showed that the micelles coated with polymer TMC could significantly improve the drug uptake of cells. TMC, because of its positive charge, interacts favorably with the negatively charged cell membrane. Like chitosan, this interaction not only promotes the absorption of TMC but also enables it to cross the cell membrane, thereby facilitating the entry of associated agents into the cell [35].

### 3.5. Precorneal Retention

Ordinarily, drugs administered to the ocular surface are rapidly cleared, with 100% of the drug removed from the surface within a maximum of 15–20 min [36]. This rapid clearance significantly curtails the precorneal residence time of the drugs and reduces their bioavailability [37]. The tear film serves as the eye’s primary line of defense. On the surface, there is a thin lipid layer, which restricts the access of aqueous formulations to the corneal interface and curtails excessive tear evaporation. Beneath the lipid layer lies the aqueous phase of the tear film, which can inactivate drugs through protein binding or enzymatic degradation, thereby decreasing the drugs’ bioavailability [38,39]. As shown in Figure 2C, different Cou-6 formulations diffused well across the corneal-conjunctival surface after administration. The Cou-6 solution was rapidly cleared from the eye within 15 min, accompanied by a significant 50% decrease in fluorescence intensity. Within 20 min, the fluorescence of C-M-D, C-M-T, and C-M-P was eliminated by 36.1%, 18.8%, and 27.7%, respectively, while the fluorescence of the Cou-6 solution was eliminated by 53.2%. This finding may be related to the cationic polymer materials in the micelles, because the more positive the charge exciting in the prepared micelle solution, the easier it is to interact electrostatically with the negative charge on the corneal surface, which is mainly caused by glycosaminoglycans (such as chondroitin sulfate and keratins sulfate) in the corneal stroma and a negatively charged phospholipid molecule on the surface of corneas carrying a large negative charge. The zeta potentials of D-M-T, D-M-P, and D-M-D were measured as 6.46 ± 0.06 mV, 6.19 ± 0.12 mV, and 6.01 ± 1.14 mV, respectively. Despite similar charge magnitudes, TMC-coated vesicles exhibited 27.1% longer corneal retention, compared to DOTAP-based micelles, and 12.3% longer corneal retention, compared to C-M-P. Several studies have indicated that the retention time also correlates with the mechanical entanglement of carriers with polymer in front of the cornea [8,40]. Therefore, the longer retention time based on TMC, compared to C-M-T and C-M-P, might also derive from the relatively higher molecule weight of TMC, which results in a combination of electrostatic and physical effects.

### 3.6. Corneal Penetration of DIO

**Figure 3:**
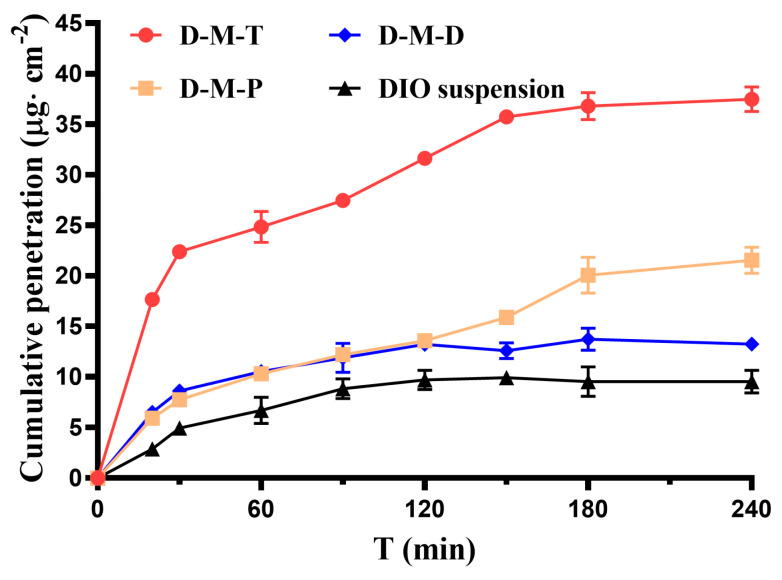
Corneal transmission curve in vitro of each preparation.

The parameters of the permeation study of the preparations are illustrated in Figure 3 and Table 1, which reveal that the DIO suspension had difficulty penetrating the cornea. The apparent permeability coefficients of D-M-T were 3.11 times and 1.49 times those of D-M-D and D-M-P, respectively. D-M-T achieved the maximum flux. D-M-T’s higher positive charge correlated with superior corneal penetration.

This suggests that while charge magnitude is necessary for mucoadhesion, polymer-specific properties (e.g., TMC’s tight junction modulation) further enhance permeability. Both D-M-P and D-M-T showed a relatively longer retention time on the surface of the rabbit cornea (Figure 2A); however, there was a significant difference between their penetration behavior. The corneal epithelium, which is the cornea’s outermost layer, acts as the limiting factor for the absorption of hydrophilic drugs. Cationic PAMAM dendrimers have been investigated as penetration enhancers that enhance the permeability of hydrophobic and hydrophilic molecules, along with various molecular weights [41]. Their absorption-enhancing effect is related to the molecular weight of hydrophilic macromolecules, and the enlargement of the pore radius at the tight junction might not have been sufficient to enable larger-sized macromolecules to pass through [42,43]. The penetration ability also exhibited a dependence on both the generation and the concentration of PAMAM. In addition to paracellular transport, PAMAM is also endocytosed. However, PAMAM G4.0-NH_2_ can be internalized nonspecifically in plasma membrane-coated vesicles [44]. As for ocular delivery, since dendrimers are large, hydrophilic molecules, a low level of penetration into the epithelium was anticipated. PAMAM G4 and G3.5 were reported to not penetrate the corneal epithelium passively to a significant extent, unless after iontophoresis [45].

The micelles coated with polymer TMC could better promote the cornea to better promote the drug to cross the cornea. Thus, it would be beneficial to improve drug bioavailability. TMC nanoparticles not only provide longer contact time with the mucosa because of their mucoadhesive nature [8], subsequently enhancing the transcorneal drug penetration [46], but also function as TMC nanoparticles by opening the tight junctions between epithelial cells, thereby allowing the paracellular transport of large hydrophilic molecules [47,48], including a redistribution of the cytoskeletal F-actin, a disrupted pattern of the transmembrane protein occludin and ZO-1, with the opening of epithelial tight junctions [49,50,51].

DOTAP is an ideal gene vector, and DOTAP-comprised cationic liposomes have been reported to penetrate deeper into the biofilms due to electrostatic interactions between the cationic liposomes and the negatively charged biofilms [52]. For the cutaneous pathway, DOTAP-comprised liposomes show a deeper penetration with more cationic charge, but only in multilamellar liposomes, as endogenous lipids exhibit negative charges to repel the penetration of liposomes [53]. We assume that under the nanoscale of the size distribution of D-M-D, with the cornea incubated within 4 h, all three preparations remained in contact with the cornea, and there was no further force between DOTAP and the corneal surface; therefore, the penetration of DIO of D-M-D was not as significant as that of D-M-T.

The hydration levels of the corneas were measured at the end of the ex vivo transcorneal permeation study to evaluate the integrity of the cornea. The values obtained were 80.2 ± 0.1%, 79.2 ± 0.3%, and 79.8 ± 0.1% for D-M-T, D-M-D, and D-M-P, respectively, indicating the relative integrity and maintenance of the vitality of the cornea before and after the experiment.

### 3.7. Corneal Penetration of Vesicles

**Figure 4:**
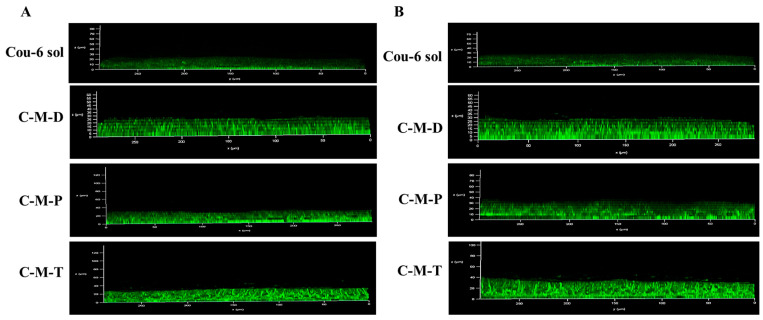
(**A**) Corneal penetration depth of different preparations at 2 h; (**B**) corneal penetration depth of different preparations at 4 h.

After proceeding for 120 or 240 min of corneal penetration experiments in vitro, cornea samples were examined using CLSM. Figure 4 shows the acquisition of optical sections (x-y plane) taken at 0 to 40 μm of successive focal planes along the z-axis of the corneal epithelia. Figure 4 shows that after 120 min of administration, the fluorescence signals from the cornea treated with C-M-P and C-M-T were detectable up to 30 μm of the cornea, significantly deeper than the Cou-6 solution and C-M-D. The trend of fluorescence penetration depths of Cou-6 solution, C-M-D, C-M-P, and C-M-T groups at 4 h was similar, indicating that the micelles coated with polymer TMC could promote most of the drug penetration through the cornea. Furthermore, TMC and DSPE-PEG-PAMAM exhibited a higher transepithelial transport and a more homogenous distribution in the deep cornea. However, by contrast, at the basal layers (around 40 μm), C-M-P showed only a few fluorescent dots, whereas many visible fluorescence clusters were seen for the C-M-T group. This could indicate a difference in the mechanisms of the TMC-coated micelles and PAMAM-composited micelles, that is, a more favorable penetration-enhancing effect of TMC. Furthermore, the smaller particle sizes of C-M-P and C-M-T facilitated the cellular uptake (Figure 2B), and the penetration mechanism might also be controlled by reactions between functional groups on the surface of the C-M-T and transmembrane proteins.

**Figure 5:**
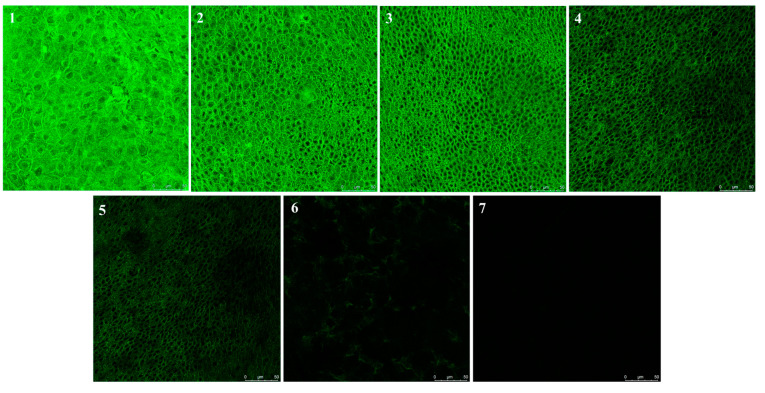
Horizontal corneal imaging of rabbit treated with C-M-T for 2 h (1–7: 5–35 μm for every 5 μm depth). Bar equals 50 μm.

To clarify the mechanism of C-M-T penetrating the corneal epithelium that could lead to this gap, Figure 5 presents a stack of horizontal sections of the corneal epithelium after a 120 min diffusion of Cou-6-coated micelles loaded with TMC, starting from the surface and moving inward into the epithelium, with images shown 5 μm apart (10–35 μm). The results of planar scanning at 2 h of C-M-T with different depths showed that the fluorescence signals were mainly distributed around the cells, and some were inside the cells, which indicated that the nano-micelles preparations could promote the drug penetrating the cornea by passing the cell.

### 3.8. Evaluation of In Vivo Efficacy

**Figure 6:**
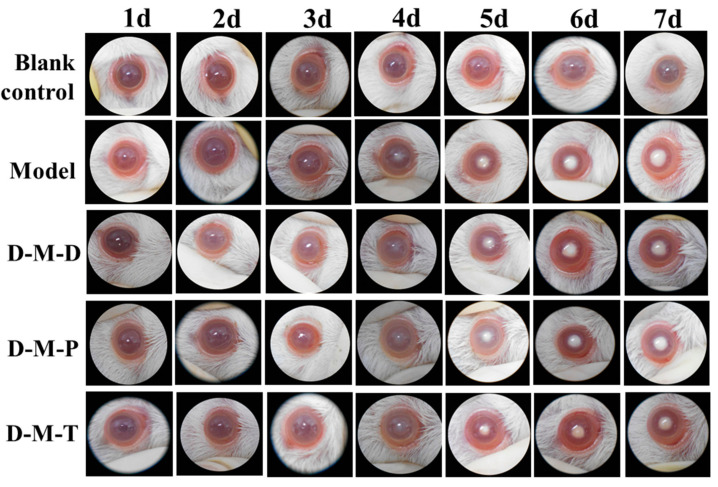
Slit lamp view of a lens with selenium-induced cataract in rats from day 1 to day 7 in each group.

All animals were examined daily by slit lamp during the experimental period. Lens opacity for animals treated with three DIO-loaded preparations and free DIO is represented in Figure 6. Compared with the blank control group, cataract genesis started in the model group on day 3 and progressed significantly in a time-dependent manner until day 7 after selenite sodium injection. Compared with the model group, the degree of lens turbidity in the three kinds of micelles administration groups was lighter and the turbidity area was smaller, indicating that the treatment of D-M-D, D-M-T, and D-M-P delayed the change of lens turbidity to a certain extent, while the delaying effect of D-M-T was more obvious. The most effective C-M-T in cataract development may derive from improved penetration and prolonged retention of TMC, and it therefore helps exhibit the antioxidant capacity of DIO.

**Figure 7:**
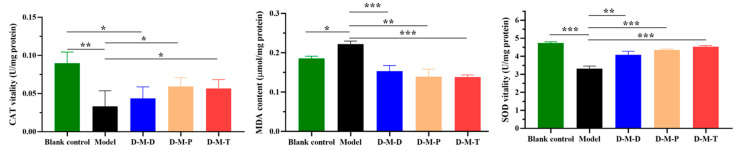
Contents of CAT, MDA, and SOD in the lens of rats in each group (***, *p* < 0.001; **, *p* < 0.005; *, *p* < 0.01).

Compared with the model group, the CAT activity in the lens of rats treated with D-M-D, D-M-T, and D-M-P increased by 24.24%, 41.58%, and 43.98%, respectively (Figure 7), which may be related to the strong antioxidant capacity of DIO. Thus, the three types of micelles influenced CAT levels in the cataract lens. Compared with the model group, the MDA values decreased by 44.60%, 66.12%, and 50.90%, respectively. The results showed that the micelles coated with TMC polymers were more effective at inhibiting the formation of lipid peroxidation products. Compared with the model group, the SOD values in the D-M-D, D-M-T, and D-M-P groups increased significantly, and the SOD in the D-M-T group was 1.11 times and 1.04 times higher than that in the D-M-D and D-M-P groups, which indicated that the three types of micelles effectively inhibited free radical oxidative damage in the lens of cataract patients and further showed that the micelles coated with polymer TMC had a stronger ability to inhibit the oxidative damage of free radicals.

Oxidative stress, which has been linked to the development of age-related cataracts, takes place when the ROS generated within or near cells surpasses the ability of normal detoxification systems to manage them. High levels of ROS can disrupt the normal structure and function of lens proteins. In addition, ROS can damage lens epithelial cells. When these cells are damaged, it may affect the normal metabolism and growth of the lens, further promoting the development of cataracts [54]. The lens does not possess blood vessels that can facilitate the dispersion of ROS. ROS are generated both during normal metabolic activities and under nonphysiological circumstances. Nevertheless, the lens is exceptionally well endowed with ROS detoxification systems. These include primary antioxidants, such as glutathione (GSH) and ascorbic acid, and enzymatic systems, such as catalase, superoxide dismutase, and glutathione peroxidase. These elements work in harmony to keep the normal adult lens in a highly reduced state [55]. Numerous studies have shown that antioxidants can protect the lens from oxidative damage and prevent the occurrence of cataracts [56,57]. CAT, a ubiquitously present enzyme, efficiently eliminates excessive hydrogen peroxide (H_2_O_2_) and has been reported to inhibit TGFβ-induced cataract-like changes in the lens [58]. MDA not only reflects the speed and intensity of lipid peroxidation to a certain extent but also combines with macromolecular proteins and small molecular nucleic acids to form insoluble products, thus accelerating lens opacity [56]. SOD catalyzes a specific disproportionation reaction involving superoxide anions, which allows SOD to effectively scavenge superoxide anions. By doing so, it helps to reduce the levels of ROS in the eye and plays a crucial role in maintaining the redox balance within the ocular tissues [59].

In the selenite-induced model group, the activities of SOD and CAT were reduced, and the level of malondialdehyde (MDA) increased compared to the model group. However, after DIO treatment, the accumulation of MDA was reduced, and the activities of SOD and CAT were enhanced. These findings suggest that DIO treatment can protect the lens from damage caused by oxidative stress. The protective effects of diosmetin on the human lens epithelial cell line SRA01/04 under oxidative stress have been evaluated [20]. Diosmetin alleviates the proliferation inhibition and apoptosis of SRA01/04 cells induced by H_2_O_2_ and UVB. It achieved this by reducing the activation of the mitogen-activated protein kinase (MAPK) pathway, which was triggered by oxidative stress.

### 3.9. Evaluation of Eye Irritation in Rabbits

Irritation of the DIO in different preparations was estimated in New Zealand big-eared white rabbits using the Draize test. After dropping D-M-D, D-M-T, and D-M-P in the right eye of rabbits, according to the Draize eye irritation response scale, the irritation scores of corneas, iris, and conjunctiva were all 0, eyes were not edematous or congestive, and no abnormality was observed.

**Figure 8:**
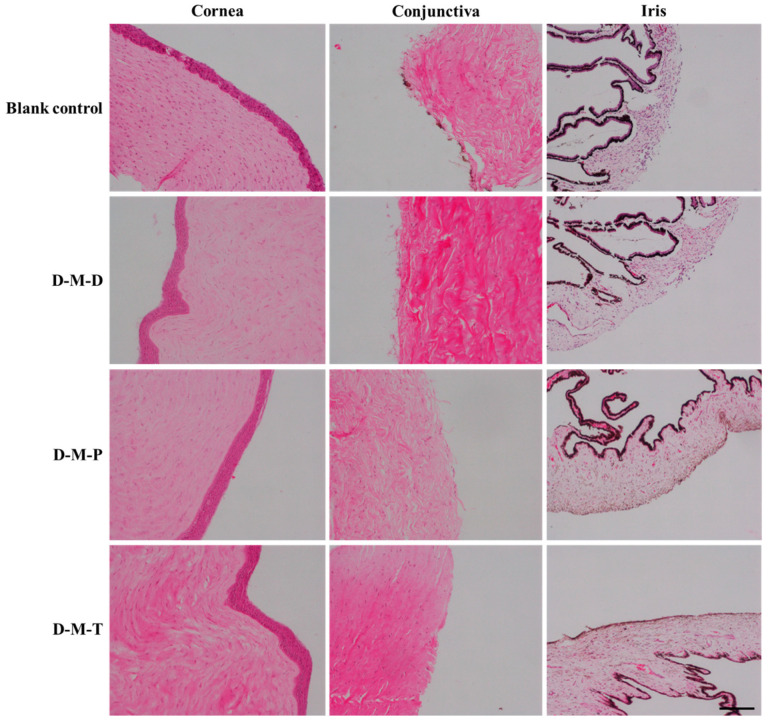
Histopathological sections of rabbit eyes in different preparation groups (Bar = 100 μm).

For the cornea, the D-M-D and D-M-T formulations showed little change compared to the blank control, indicating low irritation. In addition, the D-M-P formulation did not show any unfavorable effect on the structure of the cornea. Regarding the conjunctiva, the D-M-D formulation has a slightly disordered texture but an intact overall structure where mild irritation is possible; the D-M-T formulation has a uniform pink area and texture close to the control, suggesting little irritation; and the D-M-P formulation shows little difference from the control. As for the iris, the D-M-D formulation showed no obvious damage, the D-M-T formulation exhibited some texture changes with a relatively intact overall structure and certain possible irritations, and the D-M-P formulation had no obvious changes compared with the control (Figure 8). Overall, the structure of all tissues was intact, the cells were arranged neatly and evenly, and there was no inflammatory reaction, which further verified that D-M-D, D-M-T, and D-M-P did not result in obvious irritation in rabbit eyes at the applied concentrations.

## 4. Conclusions

In this study, three types of cationic polymers, DSPE-PEG-PAMAM, TMC, and DOTAP, and the amphiphilic materials TPGS and DSPE-PEG_2000_ were successfully prepared to encapsulate DIO polymer micelles and enhance the solubility of DIO. Because the positive charge on the surface combined with the negative charge on the corneal surface, the cationic charge of all three micelles prolonged corneal retention compared to the neutral Cou-6 solution (<36% elimination vs. 53% at the last time point). TMC-coated micelles achieved the longest retention and highest permeability (*P*_app_ for DIO = 7.28 × 10⁻^6^ cm/s), demonstrating that surface charge combined with polymer-specific mucoadhesion and penetration enhancement should synergistically improve ocular bioavailability. D-M-T exhibited better retention and higher penetration parameters, demonstrating its potential to serve as a promising drug delivery system for DIO. Further, by adjusting the levels of CAT, MDA, and SOD in the lens under oxidative stress, D-M-T shows certain advantages in attenuating the opacity induced by selenite sodium in the lens. The data from this study provide important reference material for the exploration of cataract treatment with herbal medicine.

## Figures and Tables

**Table 1:** Corneal transmission parameters in vitro of each preparation (n = 3).

Preparations	*P_app_*/10^6^·cm·s^−1^	*J_ss_*/10^3^·μg·cm^−2^·s^−1^
DIO suspension	1.68 ± 0.02	0.90 ± 0.03
D-M-D	2.34 ± 0.11	1.26 ± 0.17
D-M-P	4.87 ± 0.09	2.63 ± 0.16
D-M-T	7.28 ± 0.28	3.93 ± 0.14

## Data Availability

The original contributions presented in this study are included in the article/Supplementary Material. Further inquiries can be directed to the corresponding author.

## Supplementary Materials

The following supporting information can be downloaded at: www.mdpi.com/article/10.3390/pharmaceutics17030302/s1, Table S1: Score guidelines to calculate ocular lesions on the in vivo Draize test.
