# Supplementary material for: Influence of Different Cationic Polymer-Based Micelles on the Corneal Behavior and Anti-Cataract Effect of Diosmetin
Source: Pharmaceutics. 2025 Feb 25;17(3):302. doi: 10.3390/pharmaceutics17030302 (PMC11944416; doi:10.3390/pharmaceutics17030302)
Supplement: Supplementary file 1 [file pharmaceutics-17-00302-s001.zip › pharmaceutics-3475161-supplementary.pdf]

Supplementary materials

Table S1. Score guidelines to calculate ocular lesions on the in vivo Draize test.

Structure	Injury	Evaluation
**CORNEA**	A) Cloudiness/opacity	
	• No ulceration	0
	• Diffuse	1
	• Translucent	2
	• Opalescent	3
	• Full opacity	4
	B) Affected area	
	• None	0
	• ≤1/4	1
	• >1/4, <1/2	2
	• ≥1/2, <3/4	3
	• ≥3/4	4
**IRIS**	A) Iris injury	
	• Normal	0
	• Obvious deepening of folds, congestion, swelling, mild circumcorneal injection, and the pupil is still reactive to light	1
	• Bleeding, visible necrosis with the naked eye, and the pupil shows no reaction to light	2
**CONJUNCTIVA**	A) Hyperemia (referring only to the palpebral conjunctiva)	
	• Normal blood vessels	0
	• Hyperemia more than normal blood vessels	1
	• Diffuse hyperemia, dark-red, difficult-to-distinguish blood vessels	2
	• Diffuse hyperemia, purplish-red	3
	B) Edema	
	• None	0
	• Any swelling more than normal (including the nictitating membrane)	1
	• Obvious edema with partial ectropion of the eyelids	2
	• Eyelid more or less closed	3
	• Eyelids to be more than half-closed	4
	C) Secretion	
	• None	0
	• Secretions different from normal (excluding the small amount of secretions observed in the inner canthus of normal animals)	1
	• Secretions just making the eyelids and eyelashes moist	2
	• Periocular wetting	3

The ocular irritation index (OII) was calculated to indicate the injury degree. The ocular irritation index (OII) was calculated using the equation below.

$$OII=Corneal\ (A\cdot B\cdot 5)+Iris\ (A\cdot 5)+Conjunctiva\ (A+B+C)\cdot 2 \quad (S1)$$

A score of 0 indicates non-irritant; 0-15 shows slightly irritant; ⩾15-30 indicates moderate irritant; ⩾30-50 indicates irritant, and 50 or more shows severe irritant.
